# Dietary vitamin E and selenium supplementation improve hematological responses and productivity of growing goats consuming diluted seawater under tropical conditions

**DOI:** 10.14202/vetworld.2026.481-492

**Published:** 2026-02-10

**Authors:** Nguyen Thiet, Nguyen Thanh Dat, Nguyen Trong Ngu, Narongsak Chaiyabutr, Sumpun Thammacharoen

**Affiliations:** 1 Faculty of Animal Science, College of Agriculture, Can Tho University, 3/2 Street, Can Tho City 94000, Vietnam; 2 Hau Giang campus, Can Tho University, 3/2 Street, Can Tho City 94000, Vietnam; 3 Department of Physiology, Faculty of Veterinary Science, Chulalongkorn University, Bangkok 10330, Thailand; 4 The Academy of Science, The Royal Society of Thailand, Dusit, Bangkok 10300, Thailand

**Keywords:** antioxidant supplementation, body weight, diluted seawater, goats, hematological responses, saline water, selenium, vitamin E

## Abstract

**Background and Ai:**

Saline water use in livestock production is increasingly common in coastal and delta regions due to freshwater scarcity, but prolonged intake may compromise productivity, physiological balance, and immune function in small ruminants. Excessive intake of sodium (Na) and chloride (Cl) can induce osmotic and oxidative stress, which may disrupt hematological homeostasis and growth performance. Antioxidant nutrients such as vitamin E (VitE) and selenium (Se) can enhance cellular protection and immune resilience under environmental stress. This study aimed to evaluate the effects of dietary VitE and Se supplementation on productivity, hematological responses, and selected biochemical parameters in growing goats consuming diluted seawater (DSW) under tropical conditions

**Materials and Method:**

Ten crossbred Boer male goats (6 months old; body weight [BW] 16.66 ± 0.52 kg) were allocated to a completely randomized design with two treatments and five replicates per group over a 5-week experimental period. Goats in the control group received no supplementation, whereas goats in the treatment group received a daily premix providing 60 mg VitE and 0.9 mg Se per head. All goats consumed fresh water during period 1 (P1), followed by 1% DSW during period 2 (P2) and 2% DSW during periods 3 and 4 (P3–P4), before returning to fresh water in period 5 (P5). Dry matter intake (DMI) and water intake (WI) were recorded daily, whereas BW, plasma electrolytes, liver and kidney function indicators, and hematological parameters were measured weekly. Data were analyzed using linear mixed-effects models including treatment, period, and their interaction.

**Result:**

DMI was not significantly affected by DSW concentration or VitE–Se supplementation. WI increased at 1% DSW but declined at 2% DSW, with the supplemented group maintaining higher WI than the control group. BW change decreased in the control group during exposure to 2% DSW, whereas BW remained stable in the supplemented group. DSW increased plasma Na and Cl concentrations and elevated liver enzyme activities, while supplementation attenuated Cl accumulation and moderated Na elevation. Hematological analysis indicated greater leukocyte responses in the control group as DSW salinity increased, whereas the supplemented group maintained more stable hematological profiles. Renal and hepatic indicators remained within physiological reference ranges in both groups.

**Conclusio:**

Dietary VitE and Se supplementation mitigated DSW-associated hematological disturbances and supported WI and BW maintenance in growing goats, suggesting a practical nutritional strategy for saline water–affected production systems

## INTRODUCTION

Water scarcity is a growing global challenge, particularly in coastal regions where salinization increasingly threatens already limited freshwater availability. The use of saline water in animal production imposes substantial physiological and metabolic challenges that can adversely affect growth performance, feed efficiency, and overall health [1, 2]. Prolonged saline water consumption has been linked to dehydration, impaired nutrient digestibility, and disturbances in blood homeostasis, which may compromise animal productivity and welfare [3–5]. Increased oxidative stress induced by excessive sodium and chloride ions is a major physiological concern associated with saline water intake (WI). High-salt diets reduce antioxidant enzyme activity and increase the generation of reactive nitrogen and oxygen species [6–8]. Additionally, disruptions in electrolyte balance and hematological variables, including red blood cell (RBC) count, hemoglobin (HGB) concentration, and hematocrit (HCT), have been reported in animals consuming saline water, thereby affecting immune function and metabolic efficiency [3, 9].

Nutritional interventions to mitigate the adverse effects of saline WI are therefore essential for sustaining ruminant health and productivity. Antioxidant supplementation, including vitamin E (VitE) and selenium (Se), has been widely investigated due to its role in protecting tissues from oxidative damage and supporting immune function [10, 11]. VitE is a fat-soluble antioxidant that stabilizes cell membranes, reduces lipid peroxidation, and improves stress tolerance in animals exposed to environmental and dietary challenges [12]. Se, an essential trace mineral, is a key component of glutathione peroxidase, an enzyme involved in scavenging free radicals and maintaining redox homeostasis [13]. Mahmood *et al*. [14] reported that parenteral administration of VitE (1000 mg/kg body weight [BW]) and Se (0.3 mg/50 kg BW) alleviated saline environment-induced oxidative stress during late gestation by improving antioxidant indices, enhancing reproductive performance, and positively influencing the growth performance of suckling offspring. Our study showed that gradual adaptation to diluted seawater (DSW) at 1.5% over a 21-day period did not negatively affect goat productivity [5]. However, exposure to a higher concentration of 2% DSW within a 1-week period was associated with a reduced change in BW in sheep [15].

Although VitE and Se supplementation has been widely investigated for its antioxidant and immune-modulatory roles in livestock, most existing studies have focused on animals exposed to general oxidative stress or saline environments without controlled manipulation of drinking water salinity [10, 14]. Evidence regarding the combined effects of VitE and Se on hematological stability, electrolyte balance, and productive performance in goats consuming graded levels of DSW remains limited. In particular, there is a lack of information on whether dietary VitE and Se can mitigate hematological disturbances and BW reduction during short-term exposure to higher DSW concentrations followed by a recovery phase. This knowledge gap is especially relevant for coastal and delta regions where seasonal or intermittent use of DSW is unavoidable, and practical nutritional strategies are required to sustain goat health and productivity under such conditions.

The present study aimed to evaluate the effects of dietary VitE and Se supplementation on productivity, hematological responses, and selected biochemical parameters in growing goats consuming DSW under tropical conditions. Specifically, the study assessed whether VitE and Se supplementation could support WI, maintain BW, stabilize electrolyte balance, and alleviate hematological alterations associated with increasing DSW salinity and subsequent recovery.

## MATERIALS AND METHODS

### Ethical approval

All experimental procedures involving animals were reviewed, approved, and monitored by the Animal Ethics Committee of Can Tho University, Vietnam, under Animal Use Protocol No. CTU-AEC25018. The experiment was conducted from November 4, 2024, to February 5, 2025, at the Experimental Livestock Farm of Can Tho University (Hoa An campus), Hau Giang province, Vietnam. All procedures were performed in accordance with the Vietnamese Law on Animal Health and the Institutional Guidelines for the Care and Use of Animals in Research, and the study was reported in compliance with the **Animal Research: Reporting of** *In Vivo* **Experiments** 2.0 guidelines.

Animal handling and husbandry were designed to minimize pain, distress, and discomfort. Goats were purchased from a local farm, vaccinated according to the farm schedule, and clinically examined before enrollment to confirm good health status. Animals were housed individually in well-ventilated pens with clean flooring and were monitored daily by trained personnel. Pens were cleaned once daily, and goats received ad libitum access to natural grass and continuous access to drinking water according to the experimental protocol. Standard husbandry practices were maintained throughout the study, and all routine handling (feeding, weighing, and physiological measurements) was performed gently by trained staff to reduce stress.

Blood sampling was performed by trained personnel using jugular venipuncture under routine farm handling conditions. The volume collected (~6 mL per sampling) and the sampling schedule were selected to meet study requirements while minimizing animal burden. Animals were observed following sampling for any signs of adverse reactions, and appropriate supportive care was available if required. No invasive surgical procedures were performed, and the study design incorporated a recovery phase (P5) in which all goats were returned to fresh water to reduce potential stress associated with DSW exposure.

### Study period and location

The experiment was conducted from November 4, 2024, to February 5, 2025, at the Experimental Livestock Farm of Can Tho University, Hoa An campus, Hoa An commune, Phung Hiep district, Hau Giang province, Vietnam.

### Experimental design and animal management

Before the start of the experiment, all goats were purchased from a local farm, vaccinated according to the farm schedule, and clinically examined to confirm good health status. Animals were housed individually in well-ventilated pens with clean flooring, provided *ad libitum* access to grass, cleaned daily, and given continuous access to drinking water as per the experimental protocol. All handling, feeding, and management procedures were performed by trained personnel to minimize stress.

A total of 10 crossbred Boer male goats (6 months old; BW 16.66 ± 0.52 kg) were used in a 5-week experiment. The study followed a completely randomized design with two treatments and five replicates per treatment, consistent with previous studies [4, 5]. Goats in the control group received no supplementation, whereas goats in the treatment group were supplemented daily with a premix containing VitE (α-tocopherol acetate) and Se (sodium selenite) at 2 g/animal/day, equivalent to 60 mg VitE and 0.9 mg Se per head/day, incorporated into the concentrate feed. The premix was prepared by blending 30 g VitE and 0.45 g Se with maltodextrin to yield 1 kg of supplement.

**Figure 1:**
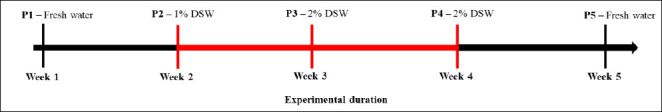
Experimental timeline of goats over five consecutive weeks under different drinking water regimes. P1 = Fresh water (days 1–7), P2 = 1% DSW (days 8–14), P3 = 2% DSW (days 15–21), P4 = 2% DSW (days 22–28), P5 = Fresh water (days 29–35), DSW = Diluted seawater.

The experiment was divided into five consecutive periods (Figure 1). During period 1 (P1), all goats consumed fresh water. During periods 2–4 (P2–P4), goats consumed DSW at concentrations of 1% (P2) and 2% (P3–P4). During period 5 (P5), goats were returned to fresh water for recovery.

### Preparation of drinking water and feeding regime

DSW was prepared weekly by diluting concentrated seawater (9.0%) with fresh water to obtain the desired concentrations using the formula C_1_V_1_ = C_2_V_2_ where C_1_ is the initial concentration, V_1_ is the initial volume, C_2_ is the final concentration, and V_2_ is the final volume. Salinity was verified using a refractometer (Master S28M, Atago, Japan). The DSW was stored in covered plastic tanks. Concentrated seawater was obtained from a local aquaculture farm.

All goats received the same diet consisting of concentrate and natural grass. The chemical composition of the concentrate feed was dry matter (DM) 89.90%, crude protein (CP) 16.90%, neutral detergent fiber (NDF) 37.80%, acid detergent fiber (ADF) 24.70%, and ash 7.20%. Natural grass contained DM 17.40%, CP 10.10%, NDF 59.70%, ADF 34.10%, and ash 10.00%. Goats were housed in individual pens (1.2 × 1.5 m) with plastic flooring, fed 300 g concentrate/day, and allowed ad libitum access to natural grass. Feeding was divided into two meals at 07:00 and 14:00 h.

### Data collection and laboratory analyses

Feed and WI were recorded daily. Feed samples from offers and refusals were collected daily and divided into two portions: one portion was dried at 105°C until constant weight to determine DM, whereas the other was stored at −20°C for chemical composition analysis. At the end of the experiment, samples were thawed, thoroughly mixed, and dried at 65°C for analysis of ash and CP [16], NDF, and ADF following the method of Van Soest *et al*. [17].

WI was recorded daily throughout the experiment. Water samples were analyzed for sodium (Na), potassium (K), calcium (Ca), and magnesium (Mg) using atomic absorption spectrophotometry (Thermo iCE 3000 series, Thermo Fisher Scientific, China). Chloride (Cl) was determined by colorimetric titration, sulfate was measured by spectrophotometry (UV-VIS 1800, Shimadzu, Japan), electrical conductivity (EC) was measured using an EC meter (Schott Instrument D-55122, Mainz, Germany), and total dissolved solids were determined using a refractometer (Master S28M, Atago, Japan). BW was recorded at the start and end of each week before morning feeding. Chemical composition of fresh water and DSW is presented in Table 1.

**Table 1:** Chemical composition of fresh water and DSW used in the experiment.

Items (g/L)	Fresh water	Diluted seawater (1.0%)	Diluted seawater (2.0%)
TDS	0.120	10.00	20.00
K	0.004	0.135	0.25
Na	0.021	3.220	6.25
Cl	0.032	7.120	13.89
Ca	0.022	0.056	0.11
Mg	0.090	0.329	0.60

Values are expressed as mean concentrations (g/L). DSW = Diluted seawater, TDS = Total dissolved solids, Na = Sodium, K = Potassium, Cl = Chloride, Ca = Calcium, Mg = Magnesium.

Rectal temperature (Tr) and respiration rate (RR) were measured at 09:00 and 15:00 h, respectively, at the end of each experimental period. Tr was measured using a digital clinical thermometer (C202, Terumo, Tokyo, Japan), and RR was determined by counting flank movements for 1 min.

At the end of each experimental week (days 7, 14, 21, 28, and 35), blood samples (~6 mL) were collected from the jugular vein at 10:00 h. Samples were divided equally: one portion was placed in heparinized tubes, kept on crushed ice, and centrifuged to obtain plasma for biochemical analysis, while the second portion was placed in ethylenediaminetetraacetic acid tubes for hematological analysis. Biochemical parameters, including urea, creatinine, aspartate aminotransferase (AST), and alanine aminotransferase (ALT), were measured using an automatic clinical chemistry analyzer (XL200, Erba Mannheim, Germany). Plasma electrolyte concentrations were determined using an electrolyte analyzer (ST200 PRO, Sensa Core, India). Hematological variables were analyzed using an automatic hematology analyzer (BHA-3000VET, Getein Biotechnology, China).

### Statistical analysis

Data are presented as means ± standard error of the mean. Data were analyzed using a linear mixed-effects model to account for repeated measurements across experimental periods:

Yijk = μ + Ti + Pj + (T×P)ij + Gk + εijk,

where Yijk represents the observed response variable, μ represents the overall mean, Ti represents the fixed effect of treatment, Pj represents the fixed effect of period (P1–P5), (T×P)ij represents the treatment × period interaction, Gk represents the random effect of goat, and εijk represents the residual error.

When significant main or interaction effects were detected, Tukey’s test was applied for post hoc comparisons. Statistical significance was declared at p < 0.05. One-way analysis of variance (ANOVA) across periods and unpaired t-tests between treatment groups were also performed.

## RESULTS

### Feed, WI, and growth performance

**Figure 2:**
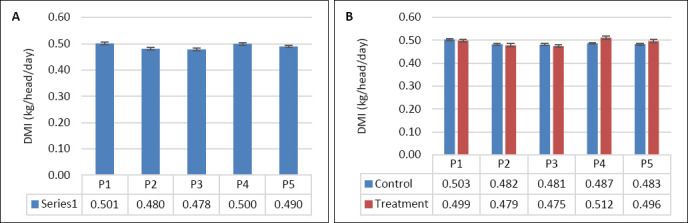
(A) Effects of vitamin E and selenium supplementation on dry matter intake (kg/head/day) across experimental periods and (B) comparison between control and treatment groups in goats consuming diluted seawater. (A) Data were analyzed by one-way analysis of variance among experimental periods. (B) Data were analyzed by an unpaired t-test between the control and treatment groups. Control = No vitamin E and selenium supplementation, Treatment = 2 g/head/day vitamin E and selenium mixture. P1 = Fresh water (days 1–7), P2 = 1% diluted seawater (days 8–14), P3 = 2% diluted seawater (days 15–21), P4 = 2% diluted seawater (days 22–28), P5 = Fresh water (days 29–35).

**Figure 3:**
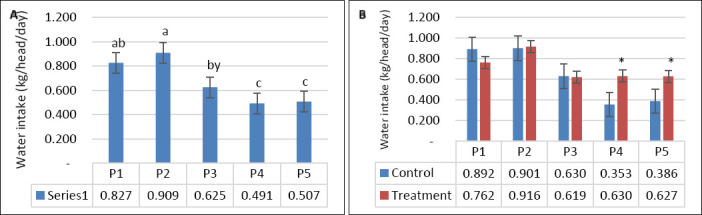
(A) Effects of vitamin E and selenium supplementation on water intake (kg/head/day) across experimental periods and (B) comparison between control and treatment groups in goats consuming diluted seawater. (A) Data were analyzed by one-way analysis of variance among experimental periods. (B) Data were analyzed by unpaired t-test between control and treatment groups. Control = No vitamin E and selenium supplementation, Treatment = 2 g/head/day vitamin E and selenium mixture. P1 = Fresh water (days 1–7), P2 = 1% diluted seawater (days 8–14), P3 = 2% diluted seawater (days 15–21), P4 = 2% diluted seawater (days 22–28), P5 = Fresh water (days 29–35). *p < 0.05, #0.05 < p < 0.10.

**Figure 4:**
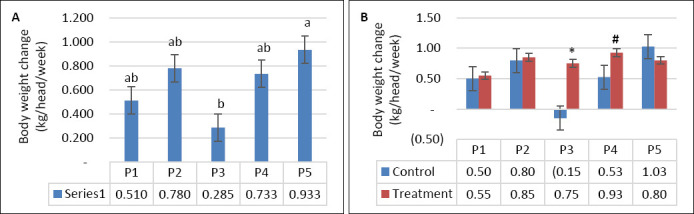
(A) Effects of vitamin E and selenium supplementation on body weight change across experimental periods and (B) comparison between control and treatment groups in goats consuming diluted seawater. (A) Data were analyzed by one-way analysis of variance among experimental periods. (B) Data were analyzed by unpaired t-test between control and treatment groups. Control = No vitamin E and selenium supplementation, Treatment = 2 g/head/day vitamin E and selenium mixture. P1 = Fresh water (days 1–7), P2 = 1% diluted seawater (days 8–14), P3 = 2% diluted seawater (days 15–21), P4 = 2% diluted seawater (days 22–28), P5 = Fresh water (days 29–35). *p < 0.05, #0.05 < p < 0.10.

DMI was not significantly affected by supplementation, seawater dilution, or their interaction (p > 0.05; Table 2, Figures 2A and 2B). In contrast, WI was significantly influenced by seawater dilution (p = 0.001) and by the interaction between supplementation and dilution (p = 0.04), whereas supplementation alone had no significant effect (p = 0.10). WI declined markedly from P1–P2 to P4–P5 (p < 0.05; Figure 3A). This reduction occurred mainly in the control group, whereas WI in the supplemented group was maintained as DSW concentration increased (Figure 3B). BW and BW change were affected by seawater dilution but not by supplementation or their interaction (Table 2). A reduction in BW change was observed during P3 and occurred predominantly in the control group, while BW change in the supplemented group remained stable (Figures 4A and 4B).

**Table 2:** Effects of DSW consumption and VitE–Se supplementation on DMI, WI, BW, BW change, RR, and Tr in goats.

Items	Control P1	P2	P3	P4	P5	Treat- ment P1	P2	P3	P4	P5	SEM	T	P	T × P
DMI (kg/head/day)	0.503	0.482	0.481	0.487	0.469	0.499	0.479	0.475	0.525	0.496	0.01	0.22	0.11	0.28
WI (kg/head/day)	0.892^a^	0.901^a^	0.630^ab^	0.353^b^	0.386^b^	0.762^a^	0.916^a^	0.619^ab^	0.630^ab^	0.627^ab^	0.07	0.10	0.001	0.04
BW (kg/head)	17.30	18.10	17.95	18.48	19.50	17.04	17.80	18.52	19.46	20.30	0.73	0.44	0.01	0.85
Change in BW (kg)	0.500	0.800	−0.150	0.525	1.025	0.520	0.760	0.720	0.940	0.840	0.21	0.12	0.04	0.11
RR – 09:00 h (breaths/min)	25	23	24	25	24	23	24	24	24	22	1	0.31	0.23	0.45
RR – 15:00 h (breaths/min)	26	25	24	25	24	24	25	25	25	23	1	0.38	0.08	0.47
Tr – 09:00 h (°C)	38.83	38.85	39.10	38.95	38.85	38.98	38.72	39.00	39.00	38.94	0.07	0.77	0.01	0.21
Tr – 15:00 h (°C)	39.13	39.22	39.35	38.88	39.05	39.28	39.06	39.14	38.98	39.02	0.10	0.66	0.02	0.32

Values are presented as mean ± SEM. a,b Different superscripts within the same row indicate significant differences (p < 0.05). DMI = Dry matter intake, WI = Water intake, BW = Body weight, RR = Respiratory rate, Tr = Rectal temperature, DSW = Diluted seawater, VitE–Se = Vitamin E and selenium, SEM = Standard error of the mean. Control = No VitE–Se supplementation, Treatment = 2 g/head/day VitE–Se mixture. P1 = Fresh water (days 1–7), P2 = 1.0% DSW (days 8–14), P3 = 2.0% DSW (days 15–21), P4 = 2.0% DSW (days 22–28), P5 = Fresh water (days 29–35).

### Plasma biochemical responses

**Figure 5:**
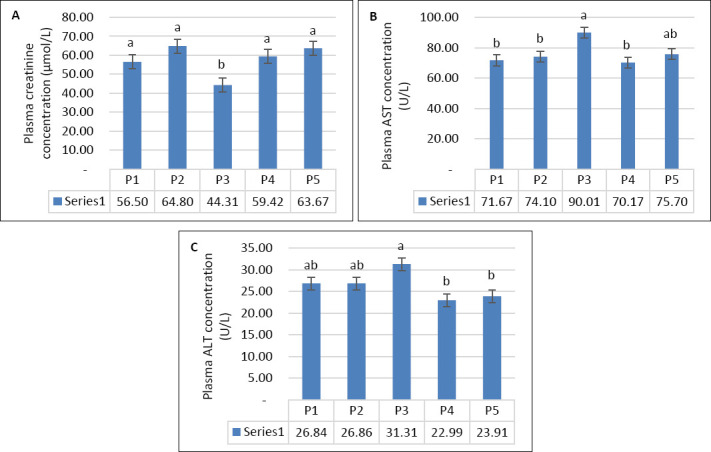
(A) Plasma creatinine concentration, (B) plasma aspartate aminotransferase activity, and (C) plasma alanine amino-transferase activity in growing goats across experimental periods while consuming diluted seawater. (A–C) Data were analyzed by one-way analysis of variance among experimental periods. P1 = Fresh water (days 1–7), P2 = 1% diluted seawater (days 8–14), P3 = 2% diluted seawater (days 15–21), P4 = 2% diluted seawater (days 22–28), P5 = Fresh water (days 29–35). *p < 0.05, #0.05 < p < 0.10.

Seawater dilution significantly affected plasma creatinine concentration (p < 0.001), whereas supplemen-tation (p = 0.14) and the interaction between the two factors (p = 0.98) had no significant effects (Table 3). When data were analyzed using one-way ANOVA, the lowest and highest creatinine values were observed during P3 and P2, respectively (Figure 5A). Hepatic enzyme activity showed a similar response pattern. Plasma AST was significantly influenced by seawater dilution (p = 0.01), but not by supplementation (p = 0.15) or their interaction (p = 0.74) (Table 3). Similarly, plasma ALT was affected by seawater dilution (p < 0.001), whereas supplementation and interaction effects were not significant (p > 0.05). The highest AST and ALT levels were recorded during P3 (Figures 5B and 5C).

**Table 3:** Effects of VitE–Se supplementation on plasma biochemical and electrolyte parameters of goats consuming DSW.

Items	Control P1	P2	P3	P4	P5	Treat-ment P1	P2	P3	P4	P5	SEM	T	P	T × P
Creatinine (µmol/L)	53.73	64.33	42.53	57.15	61.30	59.28	65.28	46.10	61.68	66.04	4.08	0.14	0.001	0.98
AST (U/L)	70.22	67.70	88.67	66.65	76.20	73.12	80.50	91.34	73.68	75.20	5.29	0.15	0.01	0.74
ALT (U/L)	27.25	26.48	32.10	23.23	24.10	26.42	27.24	30.52	22.76	23.72	1.78	0.66	0.001	0.98
Na (mmol/L)	141.47	141.87	145.50	144.77	144.07	142.38	141.84	143.34	143.20	144.28	0.61	0.17	0.001	0.085
K (mmol/L)	5.29	5.05	4.91	4.94	4.61	5.11	5.17	4.82	4.83	4.77	0.15	0.81	0.011	0.72
Cl (mmol/L)	102.27^d^	103.95^bcd^	108.10^a^	105.50^b^	104.47^bc^	102.58^cd^	103.82^bcd^	105.52^b^	103.92^bcd^	103.76^bcd^	0.43	0.001	0.001	0.012

Values are presented as mean ± SEM. a–d Different superscripts within the same row indicate significant differences (p < 0.05). AST = Aspartate aminotransferase, ALT = Alanine aminotransferase, Na = Sodium, K = Potassium, Cl = Chloride, DSW = Diluted seawater, VitE–Se = Vitamin E and selenium, SEM = Standard error of the mean. Control = No VitE–Se supplementation, Treatment = 2 g/head/day VitE–Se mixture. P1 = Fresh water (days 1–7), P2 = 1.0% DSW (days 8–14), P3 = 2.0% DSW (days 15–21), P4 = 2.0% DSW (days 22–28), P5 = Fresh water (days 29–35).

### Plasma electrolyte balance

**Figure 6:**
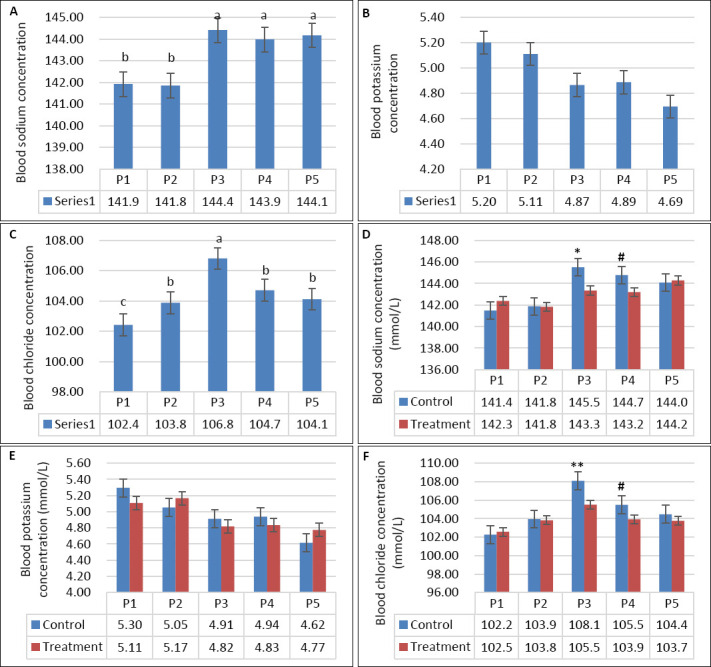
(A–C) Plasma sodium, potassium, and chloride concentrations across experimental periods and (D–F) comparison between control and treatment groups in growing goats consuming diluted seawater. (A–C) Data were analyzed by one-way analysis of variance among experimental periods. (D–F) Data were analyzed by an unpaired t-test between the control and treatment groups. Control = No vitamin E and selenium supplementation, Treatment = 2 g/head/day vitamin E and selenium mixture. P1 = Fresh water (days 1–7), P2 = 1% diluted seawater (days 8–14), P3 = 2% diluted seawater (days 15–21), P4 = 2% diluted seawater (days 22–28), P5 = Fresh water (days 29–35). *p < 0.05, #0.05 < p < 0.10.

Seawater dilution markedly influenced plasma electrolyte profiles. Plasma Na was significantly affected by seawater dilution (p < 0.001), but not by supplementation (p = 0.17) or their interaction (Table 3). Plasma Na increased progressively from P1 to P4, reflecting greater Na absorption with increasing DSW salinity (Figure 6A). Although goats returned to fresh water during P5, plasma Na remained higher than during P1, indicating sustained physiological adjustment. In addition, plasma Na in the supplemented group was lower than that in the control group during P3 (p < 0.05; Figure 6D). Plasma K was also influenced by seawater dilution (p = 0.011), showing a decreasing trend at higher salinity levels, whereas supplementation and interaction effects were not significant (p > 0.05; Table 3, Figure 6E). Plasma K decreased from P1 to P5 and peaked during P3 as DSW concentration increased (Figure 6B). In contrast, plasma Cl was affected by supplementation (p = 0.001), seawater dilution (p < 0.001), and their interaction (p = 0.012). The highest Cl level was observed in the control group during P3 and the lowest during P1 (Figure 6C). At comparable salinity levels, the supplemented group consistently exhibited lower plasma Cl, particularly during P3 and P4 (Figure 6F).

### Hematological responses

**Figure 7:**
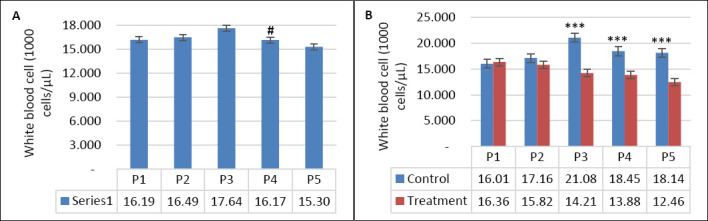
(A) Changes in leukocyte counts across experimental periods and (B) comparison between control and treatment groups in goats consuming diluted seawater. (A) Data were analyzed by one-way analysis of variance among experimental periods. (B) Data were analyzed by an unpaired t-test between the control and treatment groups. Control = No vitamin E and selenium supplementation, Treatment = 2 g/head/day vitamin E and selenium mixture. P1 = Fresh water (days 1–7), P2 = 1% diluted seawater (days 8–14), P3 = 2% diluted seawater (days 15–21), P4 = 2% diluted seawater (days 22–28), P5 = Fresh water (days 29–35). ***p < 0.001.

**Figure 8:**
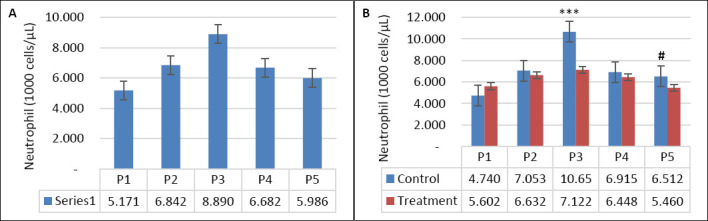
(A) Changes in neutrophil counts across experimental periods and (B) comparison between control and treatment groups in goats consuming diluted seawater. (A) Data were analyzed by one-way analysis of variance among experimental periods. (B) Data were analyzed by an unpaired t-test between the control and treatment groups. Control = No vitamin E and selenium supplementation, Treatment = 2 g/head/day vitamin E and selenium mixture. P1 = Fresh water (days 1–7), P2 = 1% diluted seawater (days 8–14), P3 = 2% diluted seawater (days 15–21), P4 = 2% diluted seawater (days 22–28), P5 = Fresh water (days 29–35). ***p < 0.001, #0.05 < p < 0.10.

Seawater dilution, supplementation, and their interaction significantly influenced hematological parameters (Table 4). WBC count was affected by seawater dilution, supplementation, and their interaction (p = 0.001). Goats consuming saline water exhibited higher WBC counts, particularly during P3 compared with other periods (p < 0.05; Figure 7A). In contrast, WBC counts in the supplemented group were maintained during DSW consumption, whereas those in the control group increased as salinity rose (p < 0.05; Figure 7B). Neutrophil counts showed a similar pattern (Figures 8A and 8B), being influenced by supplementation (p = 0.01), seawater dilution (p < 0.001), and their interaction (p = 0.001). Lymphocyte counts were significantly affected by supplementation (p = 0.001) and by the interaction between supplementation and seawater dilution (p = 0.013), whereas eosinophil counts were influenced only by seawater dilution (p = 0.02).

**Table 4:** Effects of VitE–Se supplementation on plasma biochemical and electrolyte parameters of goats consuming DSW.

Items	Control P1	P2	P3	P4	P5	Treat-ment P1	P2	P3	P4	P5	SEM	T	P	T × P
Creatinine (µmol/L)	53.73	64.33	42.53	57.15	61.30	59.28	65.28	46.10	61.68	66.04	4.08	0.14	0.001	0.98
AST (U/L)	70.22	67.70	88.67	66.65	76.20	73.12	80.50	91.34	73.68	75.20	5.29	0.15	0.01	0.74
ALT (U/L)	27.25	26.48	32.10	23.23	24.10	26.42	27.24	30.52	22.76	23.72	1.78	0.66	0.001	0.98
Na (mmol/L)	141.47	141.87	145.50	144.77	144.07	142.38	141.84	143.34	143.20	144.28	0.61	0.17	0.001	0.085
K (mmol/L)	5.29	5.05	4.91	4.94	4.61	5.11	5.17	4.82	4.83	4.77	0.15	0.81	0.011	0.72
Cl (mmol/L)	102.27^d^	103.95^bcd^	108.10^a^	105.50^b^	104.47^bc^	102.58^cd^	103.82^bcd^	105.52^b^	103.92^bcd^	103.76^bcd^	0.43	0.001	0.001	0.012

Values are presented as mean ± SEM. a–d Different superscripts within the same row indicate significant differences (p < 0.05). AST = Aspartate aminotransferase, ALT = Alanine aminotransferase, Na = Sodium, K = Potassium, Cl = Chloride, DSW = Diluted seawater, VitE–Se = Vitamin E and selenium, SEM = Standard error of the mean. Control = No VitE–Se supplementation, Treatment = 2 g/head/day VitE–Se mixture. P1 = Fresh water (days 1–7), P2 = 1.0% DSW (days 8–14), P3 = 2.0% DSW (days 15–21), P4 = 2.0% DSW (days 22–28), P5 = Fresh water (days 29–35).

Among erythrocytic indices, RBC count and HCT were not affected by treatments. However, HGB was lower in the supplemented group during P3–P5 (8.8–9.6 g/dL) compared with the control group (9.6–10.6 g/dL; p < 0.05). Mean corpuscular HGB (MCH) was also reduced by supplementation (p < 0.05), whereas no significant differences were detected in mean corpuscular volume (MCV) or mean corpuscular HGB concentration (MCHC).

## DISCUSSION

### Overall interpretation of findings

This study is the first to evaluate the synergistic effects of VitE and Se supplementation on the physiological, hematological, and biochemical responses of goats consuming DSW prepared from natural high-salinity sources. The findings demonstrate that goats receiving VitE and Se maintained DMI, hematological stability, and BW throughout the experimental period, supporting the hypothesis that antioxidant supplementation improves salt tolerance when saline water is the sole drinking source.

### Effects on feed intake and WI

DMI was not significantly affected by saline water exposure or VitE–Se supplementation, although a decreasing trend was observed as drinking water salinity increased to 2%. This observation is consistent with Zoidis *et al*. [3] and may be attributed to reduced nutrient digestibility under saline conditions, as suggested by Nguyen *et al*. [2]. Previous studies reported no effect of low saline water concentrations on DMI, whereas higher salinity levels reduced intake [3, 5]. For example, DMI remained unchanged in dairy goats consuming saline water up to 1.5% [18], while Bach Thao goats exhibited reduced DMI at similar salinity levels [5]. In the present study, goats supplemented with VitE and Se showed a smaller reduction in DMI at 2% DSW compared with non-supplemented goats, indicating that antioxidant supplementation may partially mitigate the negative effects of high-salinity on feed intake.

WI remained unchanged at 1% salinity but declined at 2% DSW, consistent with previous reports [3, 5, 15]. Animals adapt to salt stress by modulating WI, increasing renal filtration, and enhancing salt excretion [19]. In contrast, sheep adapted to saline water up to 2% exhibited a gradual increase in WI [15]. In this study, VitE–Se supplementation attenuated the decline in WI at higher salinity levels, suggesting that species, breed, and antioxidant status influence drinking behavior under saline conditions.

### Effects on BW and growth performance

BW change decreased during exposure to 2% DSW but recovered after the return to fresh water, in agreement with Nguyen *et al*. [15]. Reduced BW gain under saline conditions has been reported in goats [2, 5] and sheep [20] and is often associated with reduced DMI and impaired nutrient utilization [2, 21]. In the present study, BW reduction occurred mainly in non-supplemented goats, whereas VitE–Se–supplemented goats maintained stable BW. These findings align with Mahmood *et al*. [14], who demonstrated improved antioxidant status and growth performance in goats exposed to saline stress. The recovery phase (P5) confirmed that saline-induced BW loss was reversible, with faster and more consistent recovery observed in supplemented goats, highlighting the practical value of antioxidant support under intermittent saline exposure.

### Renal and hepatic responses

Plasma creatinine remained within normal physiological ranges despite elevations during P2 and P5, suggesting increased renal activity to maintain water–electrolyte balance under saline stress [22]. Similar findings have been reported in goats [5] and Barki sheep [23]. Elevations in AST and ALT during P3 indicate hepatic stress at 2% DSW, suggesting a threshold effect of salinity. The absence of significant differences between supplemented and non-supplemented goats implies that the dose or duration of VitE–Se supplementation was insufficient to confer marked hepatoprotection. These results agree with Abera *et al*. [9] and Ghanem *et al*. [23] but contrast with findings from Runa *et al*. [24], who reported no changes in AST or ALT at lower salinity levels.

### Electrolyte homeostasis

DSW intake disrupted electrolyte balance by increasing Na and Cl concentrations and reducing K levels. VitE–Se supplementation alleviated these disturbances, supporting electrolyte homeostasis under saline conditions. High-salt diets reduce nitric oxide (NO) availability, impair renal blood flow, and exacerbate ionic imbalance [8, 25]. VitE and Se may preserve endothelial NO synthase coupling and limit NO degradation, thereby improving renal perfusion and facilitating ion excretion. Although complete normalization of electrolytes was not achieved after the return to fresh water, supplementation promoted faster recovery. These findings are consistent with Runa *et al*. [26], and all values remained within physiological limits [22], underscoring the adaptive capacity of goats supplemented with antioxidants.

### Hematological and immune responses

WBC counts increased markedly in non-supplemented goats during P3, contrasting with earlier reports showing no effect of saline water on WBC [9]. In contrast, VitE–Se supplementation maintained stable WBC counts from P1 to P5, highlighting their immunomodulatory role under salinity-induced stress. Similar supplementation effects have not been observed in dexamethasone-stressed quail or transported heifers [27, 28], suggesting stressor-specific responses.

Elevated neutrophil, lymphocyte, and eosinophil counts in non-supplemented goats likely reflect immune activation due to disrupted homeostasis. These findings differ from those of Zoidis and Hadjigeorgiou [3] but align with evidence that environmental stress alters lymphoid tissue integrity and immune balance [29]. VitE–Se supplementation stabilized lymphocyte responses, consistent with enhanced lymphocyte counts reported in lambs [11].

Erythrocytic indices (RBC, HCT, MCV, and MCH) were largely unaffected, consistent with Yousfi *et al*. [30], although reduced HGB in supplemented goats during high-salinity exposure may reflect hemodilution [9, 31]. Despite reduced WI at higher salinity, HGB remained within physiological limits, suggesting that VitE–Se primarily modulated immune rather than hematopoietic responses under saline stress.

## CONCLUSION

This study demonstrated that exposure to 2% DSW adversely affected WI, BW change, electrolyte balance, hepatic enzyme activity, and hematological stability in goats. Supplementation with VitE and Se effectively mitigated several of these adverse effects. Goats receiving VitE–Se maintained higher WI, exhibited less reduction in BW during peak salinity (P3), showed moderated elevations in Na and Cl, and maintained more stable WBC and differential leukocyte profiles compared with non-supplemented goats. Although AST and ALT increased during high DSW exposure, values remained within physiological limits, and renal function indicators such as creatinine were not pathologically altered.

The findings indicate that dietary VitE–Se supplementation is a practical and feasible nutritional strategy to enhance goat resilience in saline water–affected production systems. This approach is particularly relevant for coastal and delta regions where DSW is used seasonally or intermittently due to freshwater scarcity. By supporting WI, stabilizing BW, and moderating electrolyte and immune disturbances, VitE–Se supplementation can help sustain productivity and animal welfare under saline stress conditions.

A major strength of this study is the use of naturally sourced high-salinity seawater diluted to controlled concentrations, providing an ecologically realistic model of saline water exposure. The experimental design incorporated graded salinity levels and a defined recovery phase, allowing assessment of both stress responses and reversibility. Comprehensive evaluation of intake, growth, biochemical, electrolyte, and hematological parameters enabled an integrated assessment of physiological adaptation to DSW.

The study was limited by a relatively small sample size and a single dose level of VitE–Se supplementation. In addition, the recovery period was short, which may not fully capture long-term normalization of electrolyte and hematological variables following saline exposure. Oxidative stress biomarkers were not directly measured, limiting mechanistic interpretation of antioxidant effects.

Future studies should evaluate dose–response relationships of VitE and Se, extend the duration of recovery periods, and include direct oxidative stress and inflammatory biomarkers to clarify underlying mechanisms. Investigations across different goat breeds, physiological stages, and longer-term production cycles would further strengthen applicability. Exploring combined nutritional strategies with other antioxidants or minerals may also provide additional benefits under saline stress.

Overall, VitE–Se supplementation improved tolerance to DSW by supporting WI, preserving BW, stabilizing electrolyte balance, and moderating immune responses in goats. These findings support the use of targeted antioxidant supplementation as a sustainable management strategy to enhance goat productivity and resilience in saline water–challenged environments.

## DATA AVAILABILITY

All the generated data are included in the manuscript.

## AUTHORS’ CONTRIBUTIONS

TN, NTD, NTN, NC, and ST: Contributed to the conception and design of the study. TN, NTD, and NTN: Contributed reagents/materials/analysis tools. TN, NTD: Performed the animal experiments. NT and ST: Analyzed the data and performed the statistical analysis. NT and ST: Wrote and revised the manuscript. All authors contributed to the final version of the manuscript and have read and approved the final version of the manuscript.

## COMPETING INTERESTS

The authors declare that they have no competing interests.

## PUBLISHER’S NOTE

Veterinary World remains neutral with regard to jurisdictional claims in the published institutional affiliations.

## References

[1] Mdletshe ZM, Chimonyo M, Marufu MC, Nsahlai IV (2017). Effects of saline water consumption on physiological responses in Nguni goats. Small Rumin Res.

[2] Nguyen T, Nguyen TN, Nguyen THN, Thammacharoen S The effects of high saline water on physiological responses, nutrient digestibility and milk yield in lactating crossbred goats. Livest Res Rural Dev. 2022a.

[3] Zoidis E, Hadjigeorgiou I (2018). Effects of drinking saline water on food and water intake, blood and urine electrolytes and biochemical and haematological parameters in goats:A preliminary study. Anim Prod Sci.

[4] Nguyen T, Nguyen VH, Nguyen TN, Thammacharoen S Effects of high-salinity in drinking water on behaviours, growth and renal electrolyte excretion in crossbred Boer goats under tropical conditions. Vet World. 2022b.

[5] Nguyen T, Nguyen TN, Chaiyabutr N, Thammacharoen S (2024a). Diluted seawater decreased weight gain and altered blood biochemical parameters in Bach Thao goats. J Appl Anim Res.

[6] Kitiyakara C, Chabrashvili T, Chen Y, Blau J, Karber A, Aslam S (2003). Salt intake, oxidative stress, and renal expression of NADPH oxidase and superoxide dismutase. J Am Soc Nephrol.

[7] Huang P, Shen Z, Yu W, Huang Y, Tang C, Du J (2017). Hydrogen sulfide inhibits high-salt diet-induced myocardial oxidative stress and myocardial hypertrophy in Dahl rats. Front Pharmacol.

[8] Zheng X, Li X, Chen M, Yang P, Zhao X, Zeng L, OuYang Y, Yang Z, Tian Z (2019). The protective role of hawthorn fruit extract against high salt-induced hypertension in Dahl salt-sensitive rats:Impact on oxidative stress and metabolic patterns. Food Funct.

[9] Abera F, Urge M, Yirga H, Yousuf Y (2025). Effect of drinking saline water on physiological, haematological and biochemical parameters of Blackhead Ogaden sheep and Somali goats. J Anim Physiol Anim Nutr (Berl).

[10] Surai PF, Kochish II, Fisinin VI, Juniper DT (2019). Revisiting oxidative stress and the use of organic selenium in dairy cow nutrition. Animals.

[11] Nurlatifah A, Astuti DA, Herdis H, Arifiantini I, Pamungkas FA, Santoso S, Diapari D, Sitaresmi PI, Setiatin ET, Diansyah AM (2025). Mitigating heat stress in Garut lambs:Synergistic effects of Lemuru fish oil, vitamin E, and selenium on antioxidant defense, hematology, and physiological responses. Vet World.

[12] Soliman EB, El-Moty KIA, Kassab AY (2012). Combined effect of vitamin E and selenium on some productive and physiological characteristics of ewes and their lambs during suckling period. Egyptian J Sheep Goat Sci.

[13] Hefnawy AEG, Tortora-Perez JL (2010). The importance of selenium and the effects of its deficiency in animal health. Small Rumin Res.

[14] Mahmood N, Hameed A, Hussain T (2020). Vitamin E and selenium treatment alleviates saline environment-induced oxidative stress through enhanced antioxidants and growth performance in suckling kids of Beetal goats. Oxid Med Cell Longev.

[15] Nguyen T, Nguyen TD, Nguyen TN, Chaiyabutr N, Thammacharoen S (2024b). Adaptation mechanism of Phan Rang sheep to salinity in drinking water under tropical conditions. Anim Sci J.

[16] Association of Official Analytical Chemists (AOAC) (1990). Official methods of analysis.

[17] Van Soest PJ, Robertson JB, Lewis BA (1991). Methods for dietary fiber, neutral detergent fiber, and non-starch polysaccharides in relation to animal nutrition. J Dairy Sci.

[18] Nguyen T, Truong KV, Nguyen KKT, Nguyen NT, Chaiyabutr N, Thammacharoen S (2024c). Effects of diluted seawater in drinking water on physiological responses, feeding, drinking patterns, and water balance in crossbred dairy goats. Vet World.

[19] Abou Hussien ERM, Gihad EA, El-Dedawy TM, Abdel Gawad MH (1994). Reaction of camels, sheep and goats with salt water. 2. Metabolism of water and minerals. Egypt J Anim Prod.

[20] Hekal FA-HA-M (2015). Homeostatic responses of sheep to salinity and heat stress conditions.

[21] Assad F, El-Sherif MMA (2002). Effect of drinking saline water and feed shortage on adaptive responses of sheep and camels. Small Rumin Res.

[22] Jackson PGG, Cockcroft PD (2002). Clinical examination of farm animals.

[23] Ghanem M, Zeineldin M, Eissa A, El Ebissy E, Mohammed R, Abdelraof Y (2018). The effects of saline water consumption on the ultrasonographic and histopathological appearance of the kidney and liver in Barki sheep. J Vet Med Sci.

[24] Runa RA, Gerken M, Riek A, Brinkmann L (2020). Boer goats physiology adaptation to saline drinking water. Res Vet Sci.

[25] Kouyoumdzian NM, Mikusic NR, Cao G, Choi MR, Penna SD, Fernández BE, Toblli JE, Rosón MI (2016). Adverse effects of tempol on hydrosaline balance in rats with acute sodium overload. Biotech Histochem.

[26] Runa RA, Maksud S, Rahman MS, Hasan M, Alam MR (2022). Impact of drinking saline water on hemato-biochemical parameters of Black Bengal goats in selected areas of Bangladesh. Saudi J Biol Sci.

[27] Uzochukwu IE, Ali LC, Amaefule BC, Okeke CC, Osita CO, Machebe NS, Yancheva V, Somogyi D, Nyeste K (2025). Impact of vitamin E and selenium supplementation on growth, reproductive performance, and oxidative stress in dexamethasone-stressed Japanese quail cocks. Poult Sci.

[28] Jung DJS, Kim DH, Baek SH, Cho IG, Hong SJ, Lee J, Lee JO, Kim HJ, Malekkhahi M, Baik M (2023). Effects of vitamin E and selenium administration on transportation stress in pregnant dairy heifers. J Dairy Sci.

[29] Hirakawa R, Nurjanah S, Furukawa K, Murai A, Kikusato M, Nochi T, Toyomizu M (2020). Heat stress causes immune abnormalities via massive damage to proliferation and differentiation of lymphocytes in broiler chickens. Front Vet Sci.

[30] Yousfi I, Salem HB, Aouadi D, Abidi S (2016). Effect of sodium chloride, sodium sulfate or sodium nitrite in drinking water on intake, digestion, growth rate, carcass traits and meat quality of Barbarine lamb. Small Rumin Res.

[31] Morsy AS, Manal MH, Gad-El-Moula DO, Hassan MS, Nagwa AA (2016). Blood picture, metabolites, and minerals of rabbits as influenced by drinking saline water in Egypt. Global J Adv Res.

